# The Spontaneous Expulsion of a Gallstone From a Cholecystocutaneous Fistula Following Percutaneous Cholecystostomy

**DOI:** 10.7759/cureus.79282

**Published:** 2025-02-19

**Authors:** Maryum Qureshi, Barnabas Goh, Paul Strauss, Masimba Nyandowe

**Affiliations:** 1 General Surgery, Austin Health, Melbourne, AUS; 2 General Surgery, Central Gippsland Health, Victoria, AUS

**Keywords:** abdominal wall discharge, calculus, cholecystocutaneous fistula, emphysematous cholecystitis, percutaneous cholecystostomy

## Abstract

We report an interesting case of a 102-year-old female who presented with a purulent abdominal discharge on the site of a previous percutaneous cholecystostomy in the setting of emphysematous cholecystitis 12 months prior. Imaging demonstrated a cholecystocutaneous fistula (CCF) and a retained calculus along the fistulous tract. On removing the drainage bag, a 2cm calculus was found at the skin level, which was also removed. The patient was discharged with oral antibiotics and found to be symptom-free at the clinical follow-up.

## Introduction

Cholecystocutaneous fistula (CCF) refers to a tract that connects the gallbladder to the skin [1,2]. CCF is generally associated with chronic cholecystitis or can occur following a surgical intervention. These fistulae are rare, and their incidence has significantly decreased thanks to advances in medical imaging enabling timely diagnosis and treatment of gallbladder pathology. CCF can present with abdominal pain, nausea, vomiting, fever, or a discharging abdominal wall site. Ultrasound (US) and computed tomography (CT) can provide a good assessment of the condition. A fistulogram can be performed for accurate delineation of the fistula tract. The management varies based on disease severity and comorbidity [1]. Nonoperative management includes antibiotics or endoscopic retrograde cholangiopancreatography (ERCP). Surgical options include laparoscopic or open cholecystectomy with excision of the fistulous tract [1-3]. We describe the case of a 102-year-old female with a gallstone emerging from her skin via a CCF a year after undergoing a percutaneous cholecystostomy.

## Case presentation

A 102-year-old female residing in a nursing home presented with upper abdominal pain and purulent discharge from the site of a previous percutaneous cholecystostomy. Her significant comorbidities included hypertension, hypercholesterolemia, type 2 diabetes mellitus, and ischemic heart disease. The patient was on clopidogrel, a non-smoker, who required assistance with activities of daily living. Two years previously, the patient had been admitted with cholangitis secondary to choledocholithiasis managed with an ERCP and stenting. A year later, the patient had been admitted with emphysematous cholecystitis complicated by a gallbladder perforation and a large, contained abscess associated with a 2cm calcified gallstone at Hartmann’s pouch.

This had been managed with intravenous antibiotics and a percutaneous transperitoneal cholecystostomy successfully. An abdominal ultrasound six weeks later had demonstrated complete resolution of the abscess. The cholecystostomy tube had been left in situ until her next clinic review 12 weeks post initial insertion, whereupon it had been noted the catheter had fallen out with complete epithelialization of the tract. Given her age and comorbidities, no further interventions had been performed. A stoma bag had been placed over the fistula site, and the patient had been discharged from the clinic.

**Figure 1:**
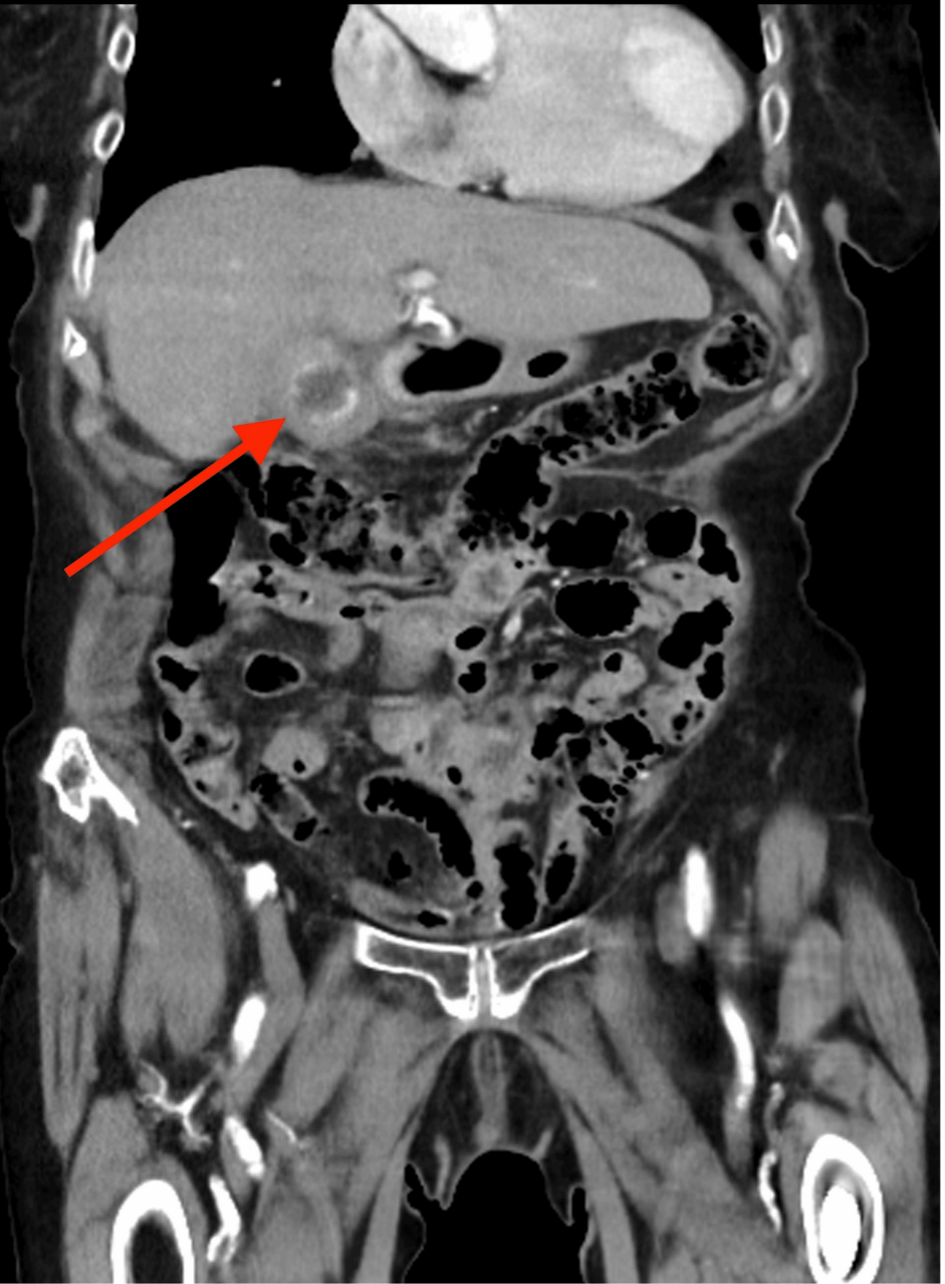
CT abdomen and pelvis (coronal view) with IV contrast demonstrating a contracted thick-walled gallbladder with a retained calculus (red arrow) CT: computed tomography; IV: intravenous

**Figure 2:**
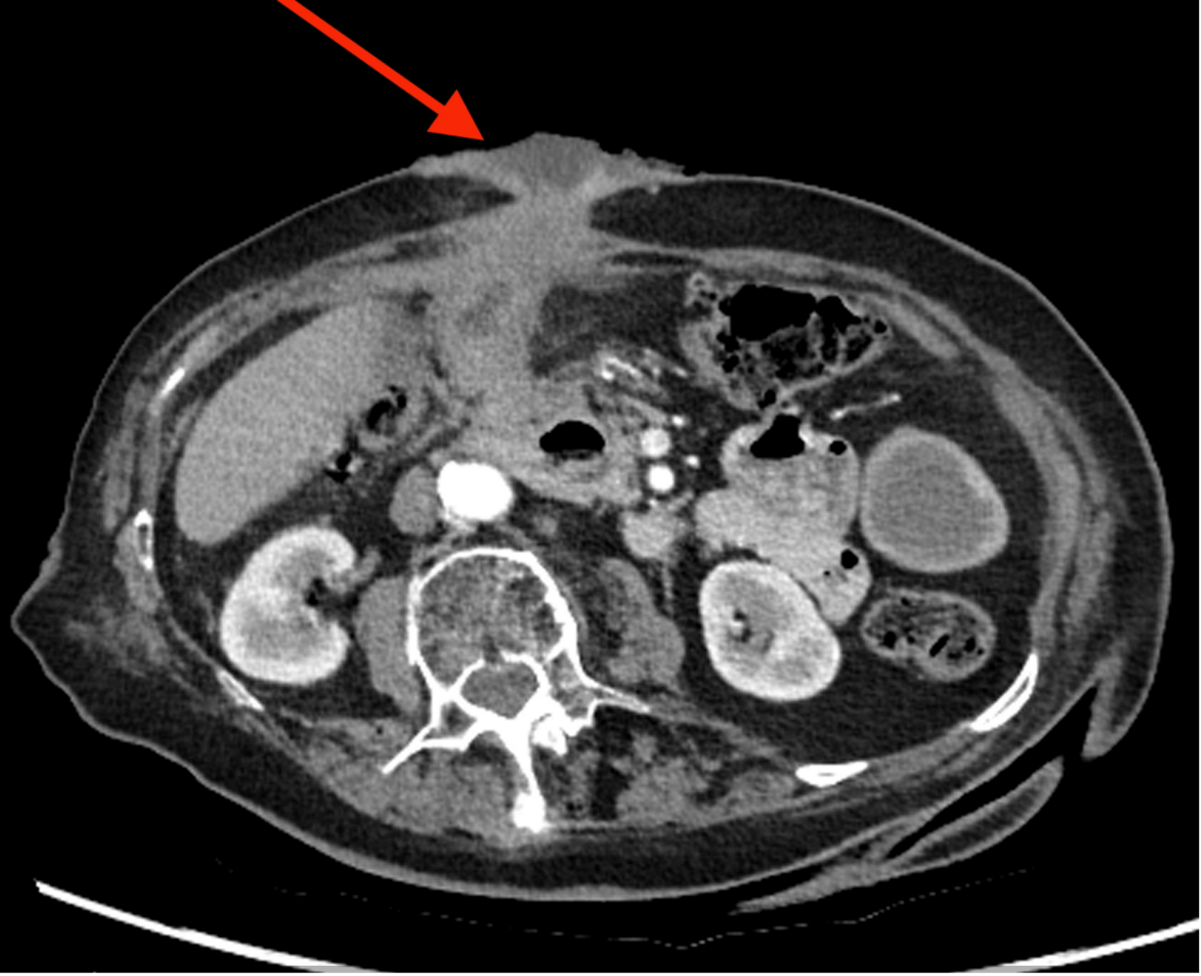
CT abdomen and pelvis with IV contrast (axial) showing a CCF along the drain tract with significant local inflammatory soft tissue thickening (red arrow) CCF: cholecystocutaneous fistula; CT: computed tomography; IV: intravenous

**Figure 3:**
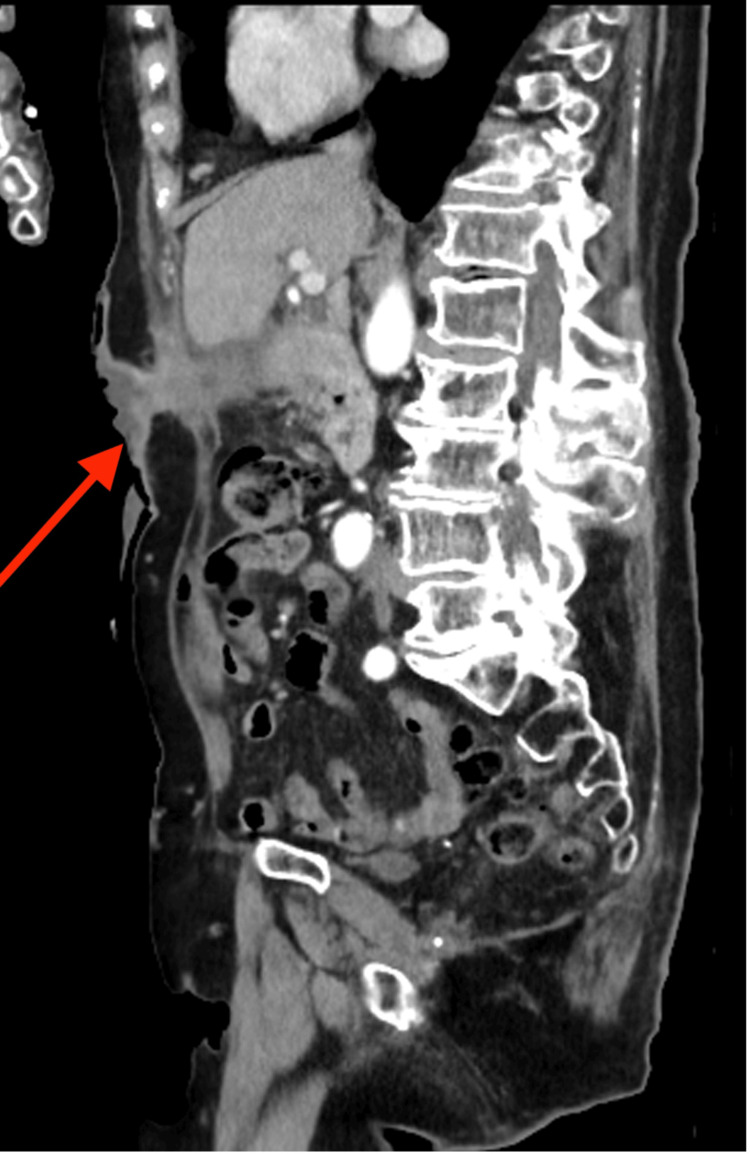
CT abdomen and pelvis with IV contrast (sagittal) showing a CCF along the drain tract (red arrow) CCF: cholecystocutaneous fistula; CT: computed tomography; IV: intravenous

The patient had continued to suffer with intermittent abdominal pain. Her main presenting complaint was purulent discharge arising from the fistula. She remained haemodynamically stable and afebrile with a mild elevation in her inflammatory markers and normal liver function tests. An ultrasound of the abdomen demonstrated a calcification at the cholecystostomy site with an apparent adjacent collection tracking toward the skin site. A subsequent CT scan showed a contracted thick-walled gallbladder with a retained calculus (Figure 1) and CCF along the drain tract with significant local inflammatory soft tissue thickening (Figures 2, 3).

**Figure 4:**
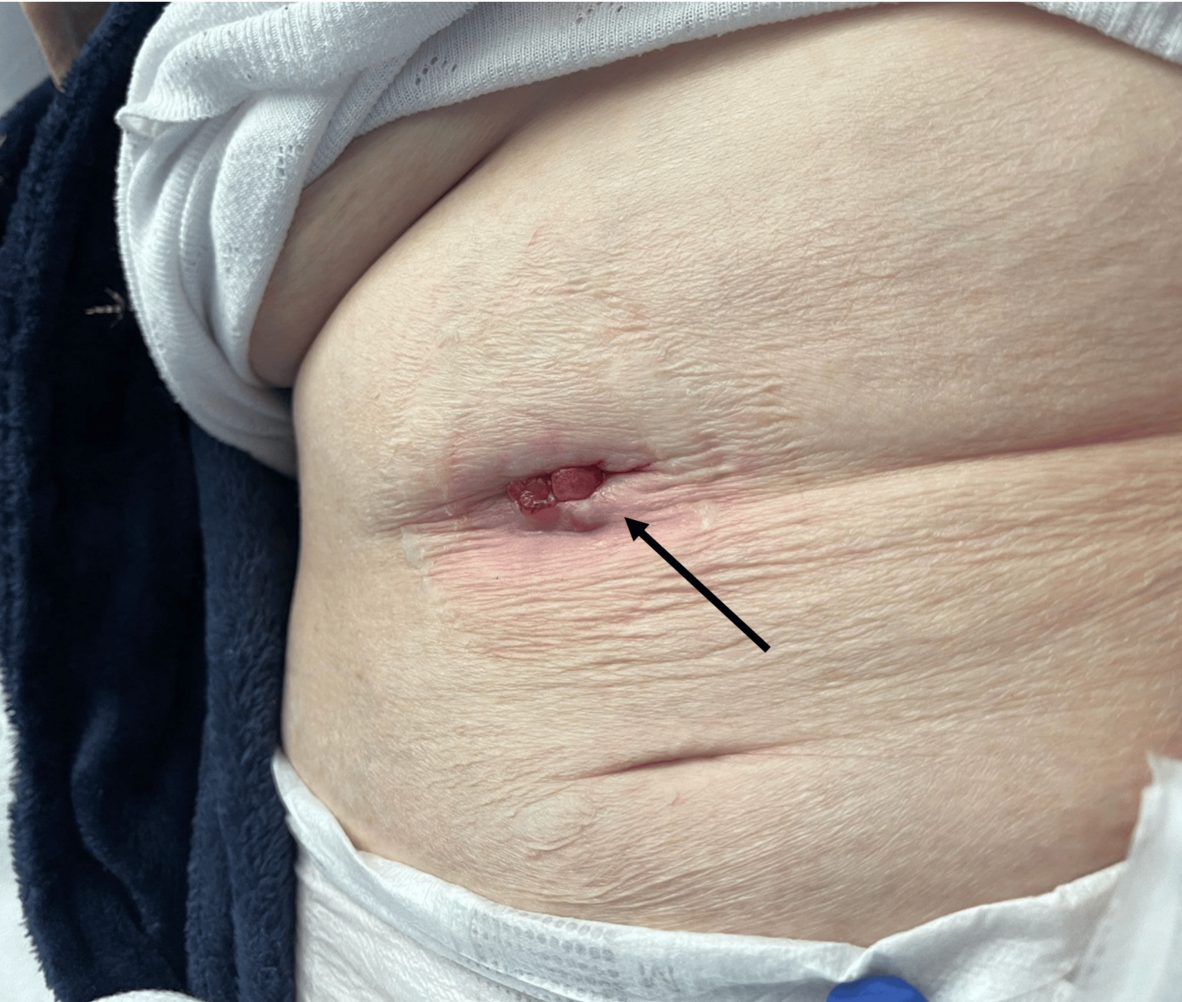
Site of emerging calculus: opening in the right upper quadrant (previous cholecystostomy drain tube site - black arrow)

**Figure 5:**
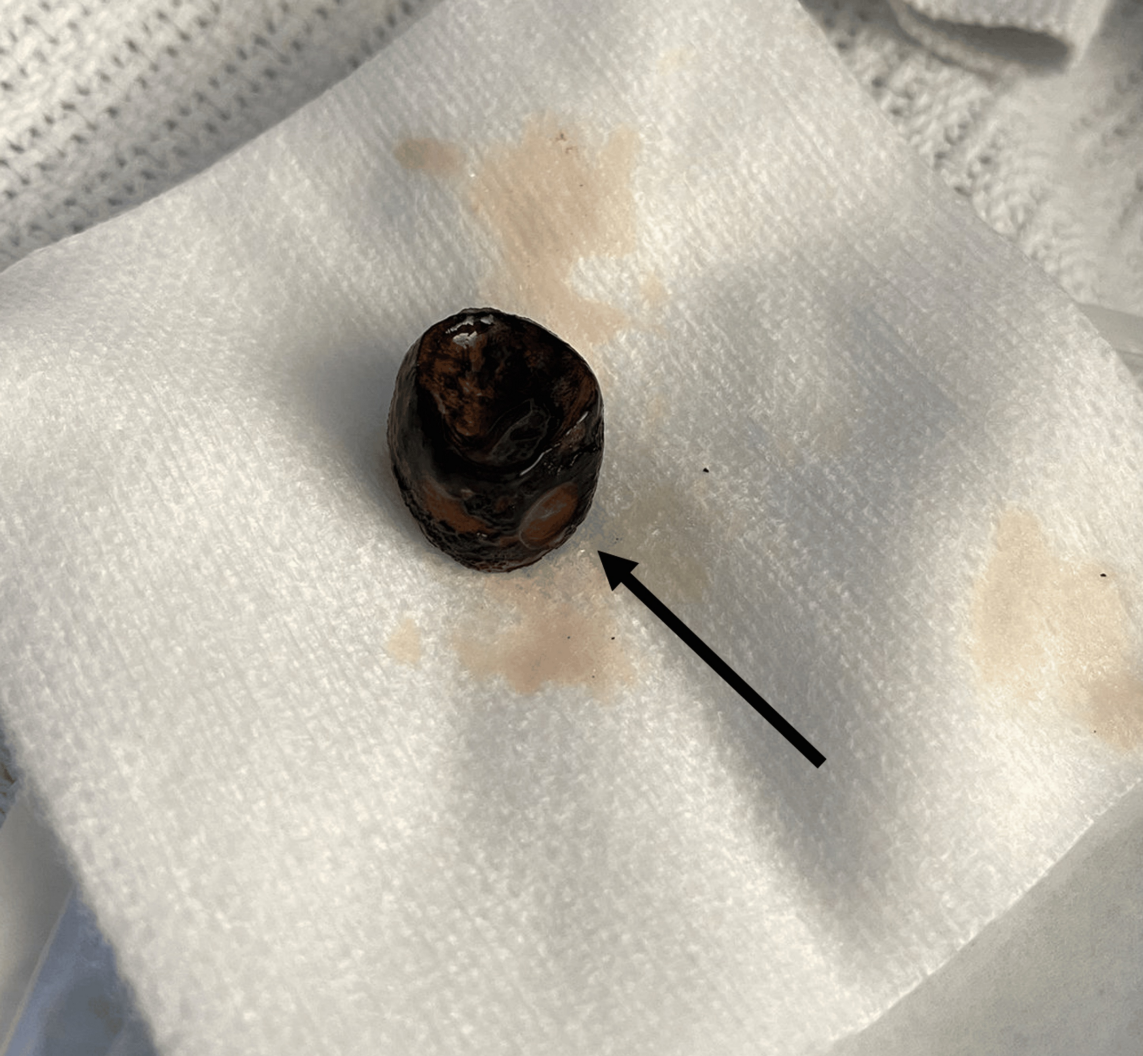
2cm calculus extracted from the cutaneous end of the fistula tract (black arrow)

On examination, a 2cm calculus had extruded from the fistula and was found at the level of the skin at the previous cholecystotomy site (Figure 4). The calculus was removed (Figure 5), and the patient was discharged with oral antibiotics.

At a follow-up clinic visit, the patient reported symptom resolution. Given the patient’s age and frailty, no further surgical intervention was deemed necessary.

## Discussion

A fistula is an abnormal connection between two epithelial surfaces. CCF is a type of biliary fistula that connects the gallbladder to the skin [1]. It can occur spontaneously with cholelithiasis and neglected chronic cholecystitis or following previous surgical intervention such as a percutaneous cholecystostomy or a subtotal cholecystectomy [1,3,4]. It is more common in females and elderly patients with incomplete treatment of gallbladder disease [1,4]. The clinical presentation of CCF is variable. Patients may present with upper abdominal pain, nausea, vomiting, fever, abdominal wall swelling, or discharge from an abdominal wall site [2,5,6]. Interestingly, our case presented with a draining skin site with extrusion of a stone 12 months post-removal of a percutaneous cholecystotomy tube.

The pathological process of CCF begins with biliary outflow obstruction which results in increased gallbladder pressure. This leads to ischemia and eventually necrosis, and perforation of the gallbladder with the development of a pericholecystic abscess. This adheres to the abdominal wall and can develop into an external fistula. In this case, a preformed tract from the insertion of a previous drain tube facilitated discharge from the diseased gallbladder through the skin creating a fistula and extrusion of a calculus through an area of least resistance [1,5,7,8].

The right upper quadrant is the most common site of a CCF opening. Other documented sites include the umbilicus, right lower quadrant, left costal margin, groin, and gluteal region [2,3,5]. Differential diagnoses include tuberculosis, chronic osteomyelitis of the ribs, pyogenic granuloma, and epidermal cysts or metastases [8]. Imaging has played a crucial role in facilitating the early diagnosis of gallbladder and biliary pathology, reducing the incidence of CCF, which was historically a common outcome of untreated gallbladder pathology [2,8]. Ultrasound and CT are essential to confirm the diagnosis. Magnetic resonance imaging (MRI) can be used to identify the fistula by demonstrating fluid within the tract. However, CT is the gold standard for diagnosis and operative planning [3,6]. A CT fistulogram can also be utilized to accurately define the fistula tract [2,6].

The management of CCF depends on patient and disease factors [1]. There is no standardized management due to the rarity of the condition. Conservative management options include antibiotics, and percutaneous drainage if associated with an abscess [1,9]. Cholecystectomy remains the definitive line of treatment for CCF. A laparoscopic surgical approach is the preferred method with excision of the fistula tract. However, an open approach may be necessary in cases with chronic inflammation, distorted anatomy, or adhesions, precluding laparoscopic surgery [1,5]. Complications of CCF include a necrotizing soft tissue infection, sepsis, or, rarely, malignant transformation of the fistula tract [2].

## Conclusions

CCF is now exceedingly rare thanks to advances in medical imaging, which have aided early diagnosis and prompt surgical management. However, it should be considered as a differential diagnosis in patients with untreated or partially treated gallbladder pathology or those with a previous surgical intervention. While management is based on individual patient factors, cholecystectomy with excision of the fistula tract is the definitive treatment modality.
